# The role of species‐specific sensory cues in male responses to mating rivals in *Drosophila melanogaster* fruitflies

**DOI:** 10.1002/ece3.3455

**Published:** 2017-10-03

**Authors:** Amanda Bretman, James Rouse, James D. Westmancoat, Tracey Chapman

**Affiliations:** ^1^ School of Biology University of Leeds Leeds UK; ^2^ School of Biological Sciences University of East Anglia Norwich UK

**Keywords:** behavioral plasticity, conspecific, *Drosophila*, heterospecific, sensory cues, sperm competition

## Abstract

Complex sets of cues can be important in recognizing and responding to conspecific mating competitors and avoiding potentially costly heterospecific competitive interactions. Within *Drosophila melanogaste*r, males can detect sensory inputs from conspecifics to assess the level of competition. They respond to rivals by significantly extending mating duration and gain significant fitness benefits from doing so. Here, we tested the idea that the multiple sensory cues used by *D. melanogaster* males to detect conspecifics also function to minimize “off‐target” responses to heterospecific males that they might encounter (*Drosophila simulans, Drosophila yakuba, Drosophila pseudoobscura,* or *Drosophila virilis*). Focal *D. melanogaster* males exposed to *D. simulans* or *D. pseudoobscura* subsequently increased mating duration, but to a lesser extent than following exposure to conspecific rivals. The magnitude of rivals’ responses expressed by *D. melanogaster* males did not align with genetic distance between species, and none of the sensory manipulations caused *D. melanogaster* to respond to males of all other species tested. However, when we removed or provided “false” sensory cues, *D. melanogaster* males became more likely to show increased mating duration responses to heterospecific males. We suggest that benefits of avoiding inaccurate assessment of the competitive environment may shape the evolution of recognition cues.

## 1 INTRODUCTION

The ability of individuals to discriminate between conspecifics and heterospecifics is key to maximizing reproductive success and avoiding potentially costly heterospecific interactions (Coyne & Orr, 2004). For example, heterospecific male–male competition can result in reproductive interference (Groning & Hochkirch, 2008). To date, heterospecific competition has been considered mostly in terms of direct contests over territories or other shared resources (Peiman & Robinson, 2010). For example, horseflies (*Tabanus* spp.,) and butterflies (*Ancyloxypha numitor*) are perceived by amberwing dragonflies (*Perithemis tenera*) to resemble conspecifics and are vigorously chased from the amberwing territories (Schultz & Switzer, 2001). Conspecific competitors are often subject to male aggression (e.g., Drury & Grether, 2014; Martin & Mendelson, 2016; Ratcliffe & Grant, 1985; Sosa‐Lopez, Martinez Gomez, & Mennill, 2016). Interestingly, the cues males use for discriminating among conspecific and heterospecific males or potential rivals may be shared with those that have evolved through female choice, for example, cues such as color patterning (e.g., in darters *Etheostoma* spp. [Martin & Mendelson, 2016], damselflies *Hetaerina americana* and *Hetaerina titia* [Drury & Grether, 2014]), and song (e.g., in Darwin's finches *Geospiza* spp. [Ratcliffe & Grant, 1985]; *Troglodytes* wrens [Sosa‐Lopez et al., 2016]). Hence male–male competition may also be important in the evolution of such cues (Grether, Losin, Anderson, & Okamoto, 2009).

However, male–male competition need not involve direct aggression. There are many examples in which males increase reproductive investment in ejaculate composition, or in behaviors such as mate guarding and copulation duration, if they perceive a high likelihood of sperm competition (Bretman, Gage, & Chapman, 2011; Wedell, Gage, & Parker, 2002). This increased investment is often costly (Bretman, Westmancoat, Gage, & Chapman, 2013). Hence males should be under selection to avoid responding in this manner to heterospecific males that pose little or no threat. Evidence to support this idea comes from male *Lygaeus equestris* seed bugs, which increase mate guarding behavior in the presence of conspecific, but not heterospecific, males (Burdfield‐Steel & Shuker, 2014). Males of numerous *Drosophila* fruit fly species tailor both their behavior and ejaculate content/investment according to the anticipation of sperm competition (Bretman, Fricke, & Chapman, 2009; Garbaczewska, Billeter, & Levine, 2013; Lizé, Doff, Smaller, Lewis, & Hurst, 2012; Mazzi, Kesäniemi, Hoikkala, & Klappert, 2009; Moatt, Dytham, & Thom, 2014; Price, Lizé, Marcello, & Bretman, 2012; Wigby et al., 2009). *Drosophila pseudoobscura* males increase mating duration following exposure to conspecific males, but not to *Drosophila persimilis* males (Price et al., 2012). *Drosophila simulans* males transfer nearly 50% more sperm to *D. simulans* females following conspecific matings, in comparison with a previous heterospecific mating by a *D. mauritiana* male (Manier et al., 2013).

To date, evidence from various species shows that chemosensory (Aragón, 2009; delBarco‐Trillo & Ferkin, 2004; Carazo, Font, & Alfthan, 2007; Lane et al., 2015; Thomas & Simmons, 2009) and acoustic (Bailey, Gray, & Zuk, 2010; Gray & Simmons, 2013) cues can be used by males to assess the level of conspecific sperm competition. Territorial competitors tend to be recognized through the detection of multiple cues within the same, or different, sensory modalities (Grether, 2011), and multimodal cues can also be used in the evaluation of sperm competition threat. For example, in *D. melanogaster*, any paired combination of sound, smell, and touch is required in order for males to respond to a conspecific rival (Bretman, Westmancoat, Gage, & Chapman, 2011), and manipulations of single sensory cues slow the speed with which *D. melanogaster* males can swap between high and low sperm competition modes (Rouse & Bretman, 2016). Conflicting data suggest that male *D. melanogaster* require only visual cues, specifically the perception of the red eyes of another fly, in order to respond to *D. simulans* or *D. virilis* males as rivals (Kim, Jan, & Jan, 2012). *Drosophila pseudoobscura* males require both olfactory and tactile cues to respond to conspecific rivals, whilst vision is unimportant (Maguire, Lize, & Price, 2015).

We suggest that the complex cues may be used to carry multiple types of information: whether the rival fly is male, a conspecific and present for a sufficiently long period to represent a threat. The natural context of such signaling suggests that conspecific recognition within mixed‐species groups may be important, as Drosophilids can be found in mixed‐species groups in the wild (Atkinson, 1979). Within such groups, hybrid matings may also occur, and there are varying degrees of pre‐ and postzygotic isolation (Coyne & Orr, 1989, 1997).

We tested the idea here that multimodal cues convey information that enable *D. melanogaster* males to avoid making erroneous sperm competition responses to heterospecific males. The predictions are not straightforward, because heterospecifics that are infrequently encountered might elicit greater rivals’ responses than for closely related species with which *D. melanogaster* can hybridize, because allopatry minimizes selection for heterospecific discrimination (e.g., Magurran & Ramnarine, 2004; Wellenreuther, Tynkkynen, & Svensson, 2010). Hence, a lack of response could be driven either by males being unable to distinguish conspecifics from heterospecifics, or because insufficient cues are present to prompt a sperm competition response.

In order to confirm that we were manipulating the important sensory modalities, we first examined whether strain differences could explain conflicting reports on the role of visual cues in rivals’ responses within *D. melanogaster* (Bretman, Westmancoat et al., 2011; Kim et al., 2012). We then tested whether *D. melanogaster* males responded to males of four other species when exposed to a full sensory repertoire from the heterospecifics or when single sensory cues were removed in turn (Bretman, Westmancoat et al., 2011). This enabled us to test two predictions: (1) Given a full sensory repertoire, males should avoid investing in “rivals’ responses” to heterospecifics that pose no sperm competition threat and (2) the sensory modalities used to convey species‐specific information can be identified by manipulating cues in order to “trick” males into responding to heterospecific rivals as they would to conspecifics.

## 2 MATERIALS AND METHODS

### 2.1 Choice of test species

We chose a range of species as heterospecific rivals (*D. simulans, yakuba, pseudoobscura,* and *virilis*)*. Drosophila melanogaster* shared its last common ancestor with *D. simulans* ~ 5 MYA, with *D. yakuba* ~ 13 MYA, with *D. pseudoobscura* ~ 55 MYA, and with *Drosophila virilis* ~ 63 MYA (Tamura, Subramanian, & Kumar, 2004). In terms of geographical range, *D. melanogaster* and *D. simulans* are cosmopolitan species, although ancestrally originating from Africa (Lachaise & Silvain, 2004). *D. yakuba* is widespread in Africa, and *D. pseudoobscura* is found across North America, and *D. virilis* in North America and East Asia (Ashburner, Carson, & Thompson, 1981). Contemporary populations of *D. melanogaster* can come into contact with all of the species tested here, although they will mate only with *D. simulans*, resulting in viable but sterile hybrids (Sturtevant, 1920). When females are multiply mated, conspecific sperm outcompete heterospecific sperm (Price, 1997), a process influenced by seminal fluid proteins (Castillo & Moyle, 2014).

### 2.2 Fly stocks and husbandry

Wild‐type *Drosophila melanogaster* were from a large laboratory population originally collected in the 1970s in Dahomey (Benin). This strain was used in our previous, related studies (e.g., Bretman et al., 2009). For the visual cues experiment, *D. melanogaster* of the Canton‐S wild‐type strain were also used (supplied by Dr Tom Price). *D. simulans*,* D. yakuba,* and *D. virilis* were obtained from the San Diego Stock Center. *Drosophila pseudoobscura* was derived from 100 females collected from a natural population Arizona, USA, in 2008 by Dr Tom Price. *D. melanogaster* were maintained on standard sugar‐yeast medium (100 g brewer's yeast powder, 50 g sugar, 15 g agar, 30 ml Nipagin (10%w/v solution), and 3 ml propionic acid, per liter of medium).

Rearing of *D. melanogaster* and all experiments were conducted in a 25°C humidified room, with a 12:12 hr light:dark cycle. Other species, and all males for the period of exposure to rivals, were maintained in an incubator at 22°C (within the optimal range for all species) under a 12:12 light:dark cycle. *Drosophila melanogaster* larvae were raised at a standard density of 100 per vial, supplemented with live yeast liquid. The other species used in the study did not lay well onto our standard grape juice egg collecting medium; hence, to standardize larval density for those, we placed parents of the experimental males in groups of five females and five males per vial for successive periods of 48 hr. To account for differences in development and maturity, *D. virilis* and *D. pseudoobscura* were 3–5 days old, and *D. simulans* and *D. yakuba* 1–2 days old, when treatment vials were set up.

### 2.3 Measurement of mating duration

At eclosion, sexes were separated using ice anesthesia and stored 10 per vial in single‐sex groups. On the day after eclosion, *D. melanogaster* males were assigned randomly as rival or focal males. Males from other species were used as rival males and given an identifying wing clip using light CO_2_ anesthesia, a procedure that does not affect the response of *D. melanogaster* focal males to rivals (Bretman, Westmancoat et al., 2011). Focal males were then held on their own or exposed to a rival for 3 days. On the 5th day after eclosion for *D. melanogaster*, mating tests were conducted. In these, focal *D. melanogaster* males were introduced singly to a female wild‐type *D. melanogaster* each, and allowed 2 hr to mate. Final sample sizes for all experiments are given in Table 1.

**Table 1:** Sample sizes for each treatment of each experiment. The first is the experiment in which visual cues were manipulated in two strains of *Drosophila melanogaster* wild types (Canton‐S and Dahomey). Next are the sample sizes for the three replicate experiments in which *D. melanogaster* (“*mel”*) focal males were exposed to conspecifics or heterospecific males of each of *Drosophila simulans* (“*sim*”), *Drosophila yakuba* (“*yak”*), *Drosophila pseudoobscura* (“*pse”*), or *Drosophila virilis* (“*vir”*), with no manipulation of sensory cues. The remainder of the table shows the sample sizes for the corresponding experiments in which the auditory, tactile, and olfactory cues present for the *D. melanogaster* focal males exposed to conspecific and heterospecific males were manipulated as indicated. CHCs = cuticular hydrocarbons

Experiment	Rival exposure treatment		
	Mirror down	Mirror up	Plus rival
Visual cues in response of *D. mel* to conspecifics			
Canton‐S	34	33	33
Dahomey	36	30	30

	No rival	*mel*	*sim*	*yak*	*pse*	*vir*
Responses of *D. mel* to conspecifics and heterospecifics						
Unmanipulated sensory cues						
Experiment 1	39	40	36	37	34	38
Experiment 2	24	25	24	22	25	26
Experiment 3	46	45	44	44	37	45
Auditory cues manipulated						
Nonfocal male wings removed	36	37	36	34	31	35
Focal males carrying *inactive* mutation	26	27	28	36	30	30
Tactile cues manipulated						
Separated by netting	36	38	33	31	40	36
Olfactory cues manipulated						
Focal male carrying *Orco* mutation	34	28	32	29	29	27
Focal male 3rd antennal segment removed	36	34	31	29	30	31
CHCs added						
Hexane carrier control	29	34	22	20	22	31
CHC wash treatment	34	35	34	25	27	33

### 2.4 The role of visual cues in *D. melanogaster* sperm competition responses to rivals

In order to inform our experimental design, we first assessed whether conflicting data on the role of visual cues in responses to conspecific rivals within *D. melanogaster* (Bretman, Westmancoat et al., 2011; Kim et al., 2012) could arise from strain differences. To do this, we replicated the same design as Kim et al. (2012), in which mirrors were used to simulate the presence of a rival male, and tested the Dahomey (Bretman, Westmancoat et al., 2011) and Canton‐S (Kim et al., 2012) wild‐type genetic backgrounds. For each strain, we used two treatments in which mirrors (12 mm diameter) were placed at the bottom of a vial with a single male, either mirror side up (to simulate the presence of a rival) or mirror side down (as a control). To ensure that both strains responded as expected to conspecific rivals, we included a positive control treatment in which each male was exposed to a conspecific male from their own same strain. All males were then given the opportunity to mate in a mating test, as above, to a female of their own strain.

### 2.5 Responses of *D. melanogaster* to conspecific and heterospecific rivals

We tested the mating duration responses of *D. melanogaster* males following 3 days of exposure to heterospecific males in control and sensory manipulated conditions. Each experiment contained a control treatment of *D. melanogaster* males held singly (no rival) and then five “rival” treatments (each focal *D. melanogaster* male exposed one “rival” of *D. melanogaster*,* D. simulans*,* D. yakuba*,* D. pseudoobscura,* or *D. virilis*). After these exposures, focal male mating duration was measured. We first conducted three replicate experiments in which there was no manipulation of sensory cues. The aim was to establish whether *D. melanogaster* males would consistently respond to a heterospecific rival as they would to a male of their own species (i.e., whether they would subsequently mate for significantly longer than in the “no‐rival” negative control treatment).

We next manipulated sensory cues (auditory, tactile and then olfactory) in separate experiments for each of the six exposure treatments (single males and five “rival” exposed treatments). We manipulated auditory cues by either removing the wings of rival males entirely, so they could not produce song, or using a hearing‐defective focal male (*D. melanogaster* carrying the *inactive* mutation [Gong et al., 2004]). To remove tactile cues, we separated males from rivals by using porous netting. To test olfactory cues, we used focal mutant males lacking *Orco* (formally *Or83b*, a coreceptor necessary for odorant perception in toto [Larsson et al., 2004]) or wild‐type focal males from which we had removed the third segment of the antennae, which contains sensillae bearing the odorant receptors required for males to respond to the odors of other flies (van der Goes van Naters & Carlson, 2007). Finally, we tested whether *D. melanogaster* males could be tricked into responding to males of all the heterospecific species equally, following exposure to false olfactory cues. To do this, we exposed all focal males to *D. melanogaster* male cuticular hydrocarbons (CHCs) extracted in hexane, using a hexane only treatment as a negative control. CHCs were extracted by immersing 50, 5‐day old males in 1 ml of hexane for 30 min (Bretman, Westmancoat et al., 2011).

### 2.6 Statistical analysis

Analyses were carried out in R v 3.3.1. No dataset conformed to normality for all treatment groups; hence, medians are presented rather than means. Some skew and kurtosis was observed. However, this was not consistent, and none of the distributions were bimodal. The error structures employed to account for such effects are described for each dataset, below. The visual cues experiment data were analyzed using a GLM with quasi Poisson errors (to account for underdispersion), with strain and rival treatment designated as fixed factors. We then used analysis of deviance (AoD) to remove terms in order to achieve minimal, simplified statistical models. Differences between the two strains in the visual cues experiment were then compared using a Mann–Whitney *U* test, and the effect of rival treatment (single, single plus mirror, paired) was analyzed using post hoc Tukey's pairwise comparisons (with Bonferroni correction for multiple comparisons). For the three replicate experiments using unmanipulated heterospecific “rivals,” we performed a GLMM with rival treatment as a fixed factor and replicate experiment (block) as a random factor. We tested this against a null model, with only the random effect of block, using AoD. We then compared rival treatment groups using post hoc Tukey's pairwise comparisons (with Bonferroni correction for multiple comparisons). All other experiments, except the CHC addition, were analyzed using Kruskal–Wallis (KW) tests with post hoc tests as before. For the CHC addition experiment, we used a GLM with quasi Poisson errors (to account for underdispersion), with rival treatment and CHC/hexane treatment as fixed factors, and reduced to the minimal model using AoD, followed by post hoc tests as before.

## 3 RESULTS

### 3.1 The role of visual cues in *D. melanogaster* sperm competition responses to rivals

**Figure 1:**
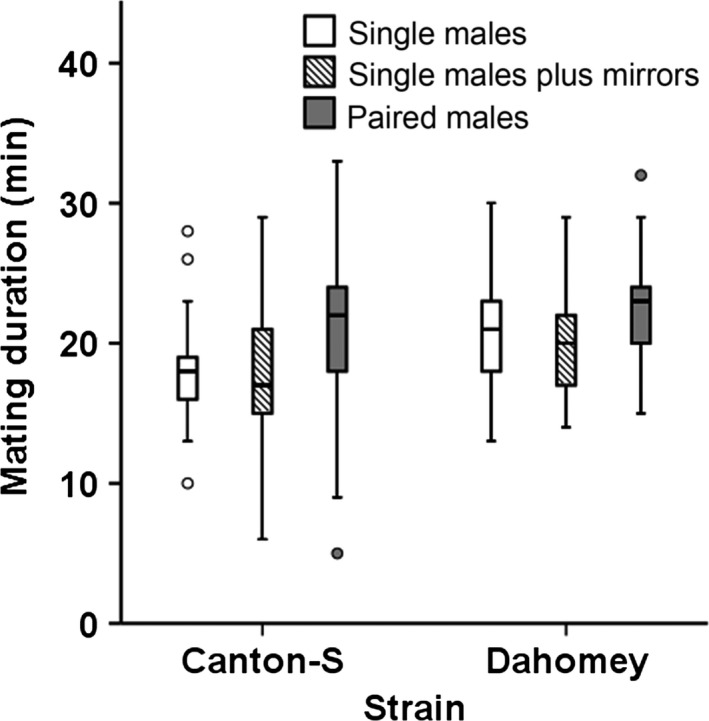
Mating duration responses of two strains of *Drosophila melanogaster* (Canton‐S and Dahomey wild types) with wild‐type females of their own strain, following simulated or actual exposure to visual cues of competition from conspecific rivals. Males of each strain were held singly in vials against a mirror with the reflective side up (“single males plus mirrors,” to simulate the presence of a conspecific rival through visual cues) or down (“single males” control) or paired with a conspecific male (“paired males”)

Both Dahomey and Canton‐S strains responded in the same way to rivals (no significant interaction between strain and rivals treatment, AoD *F*_1, 203_ = 0.629, *p *=* *.429; Figure 1). Paired males that were exposed to rivals mated for significantly longer than did either single males (*p *=* *.006) or single males with a mirror (*p *<* *.001). There was no significant difference between the mating duration of single males (no rivals) with or without mirrors (*p *=* *.964). Overall, Dahomey flies mated for significantly longer than Canton‐S flies (Mann–Whitney *U* test = 6947.500, *N* = 207, *p *<* *.001). The results are consistent with those of Bretman et al. (2011, 2011b) and show that across two different strains, visual cues alone were not sufficient for *D. melanogaster* males to detect and respond to conspecific rivals by extending mating duration. These results informed our decision not to manipulate vision in our investigation of sensory cues in species‐specific information.

### 3.2 Responses of *D. melanogaster* to conspecific and heterospecific rivals

**Figure 2:**
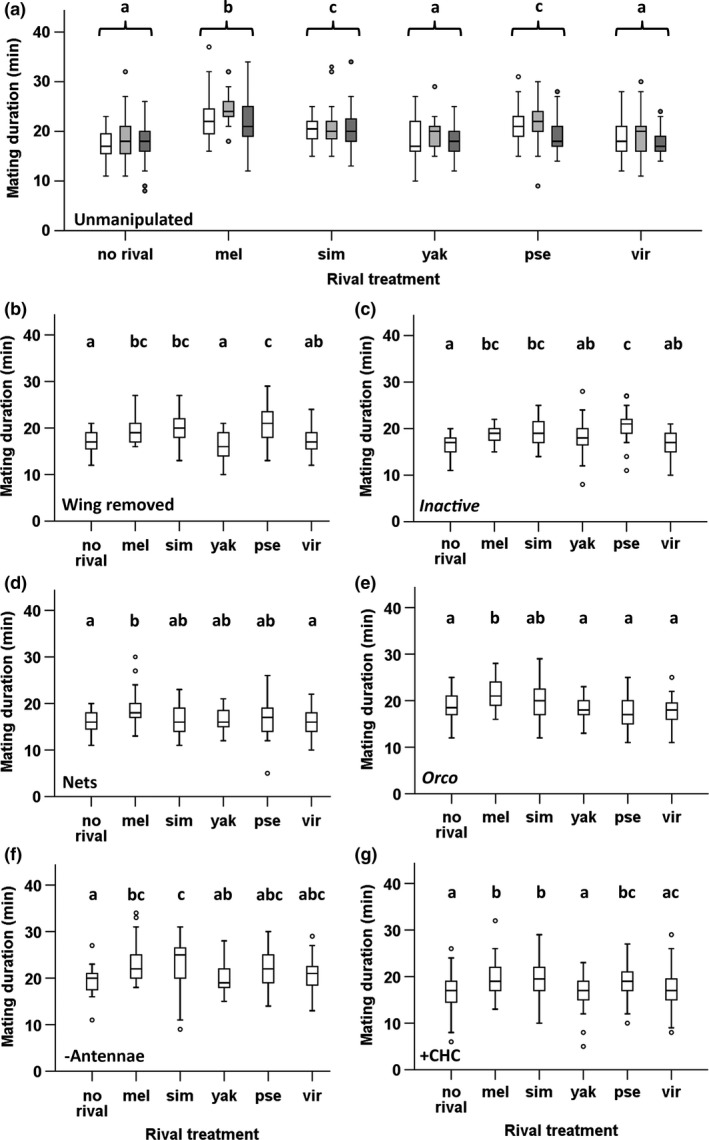
Mating duration responses of *Drosophila melanogaster* focal males to conspecific or heterospecific rivals. In each experiment, males were maintained on their own (no‐rival) or exposed to a “rival” for three days prior to mating. *D. melanogaster* (mel), *Drosophila simulans* (sim), *Drosophila yakuba* (yak), *Drosophila pseudoobscura* (pse), or *Drosophila virilis* (vir). (a) Mating duration responses of *mel* focal males to *mel*,* sim*,* yak*,* pse,* or *vir* “rivals” males—no manipulation of sensory cues. Three replicated experiments are shown (1—white, 2—light gray, 3—dark gray). (b) Mating duration responses of *mel* focal males following manipulation of auditory cues—by removing the wings of *mel*,* sim*,* yak*,* pse* or *vir* “rival” males, or (c) using hearing‐defective focal *mel* males carrying the *iav* mutation. (d) Mating duration responses of *mel* focal males following manipulation of tactile cues, by maintaining *mel* males in vials separated from *mel*,* sim*,* yak*,* pse* or *vir* “rival” males by netting. (e) Mating duration responses of *mel* focal males following manipulation of olfactory cues—using olfactory‐defective focal *mel* males carrying the *Orco* mutation, or (f) by removing the third antennal segment of wild‐type *mel* focal males, or (g) by providing *mel* male CHCs extracted in hexane (data shown are CHCs combined with the carrier control as the effect of the addition of CHCs was nonsignificant). Treatments that do not share a letter were significantly different (post hoc Tukey's tests with Bonferroni adjustment)

We found a significant effect of exposure treatment (i.e., species identity) on subsequent mating duration of focal *D. melanogaster* males across the three replicate experiments (AoD $\chi_{5}^{2}=87.562$, *p *<* *.0001; Figure 2a). *D. melanogaster* males exposed to a conspecific male extended mating duration significantly, as expected based on previous studies. In contrast, *D. melanogaster* males exposed to *D. yakuba* or *D. virilis* “rivals” did not differ in mating duration in comparison with the no‐rival treatment. Interestingly, following exposure to *D. simulans* or *D. pseudoobscura* rivals, *D. melanogaster* males significantly increased mating duration over the no‐rival treatment. The extension of mating duration was of “intermediate” duration in comparison with the responses of *D. melanogaster* males to conspecific rivals. Absolute mating duration (and the difference between ± rival treatments) differed between experiments. To account for this, we summarized the results in terms of standardized differences (Table 2). For this, we expressed the response of focal *D. melanogaster* males to a heterospecific “rival” as a proportion of the response to a conspecific rival ([+heterospecific rival median] – [no‐rival median])/([+conspecific rival median] – [no‐rival median]). This analysis showed that the extended mating duration responses of *D. melanogaster* males following exposure to *D. simulans* or *D. pseudoobscura* males were about half of that observed following exposure to a conspecific rival.

**Table 2:** Summary of standardized mating duration responses of *Drosophila melanogaster* males following exposure to heterospecific males. Heterospecific responses of *D. melanogaster* focal males as a proportion of conspecific responses ([median + heterospecific rival] − [median no‐rival])/([+conspecific rival median] – [median no‐rival]). A value of 1 indicates that the *D. melanogaster* focal males responded to a heterospecific rival to the same extent as they did to a conspecific rival. Blue boxes highlight instances where the manipulations resulted in an increase, and orange boxes a decrease, of at least 0.5 compared to the unmanipulated control (i.e., a significant mating duration response following exposure to a heterospecific male). *D. simulans* (*D. sim*), *D. yakuba* (*D. yak*), *D. pseudoobscura* (*D. pse*), or *D. virilis* (*D. vir*). Auditory, tactile, and olfactory sensory manipulations were as described in the text, CHC = cuticular hydrocarbon

Sensory modality manipulated	Type of manipulation	“Rival” male			
		*D. sim*	*D. yak*	*D. pse*	*D. vir*
None		0.4	0	0.4	0
Auditory	Wing removed	1.5	−0.5	2	0
	*iav*	1	0.5	2	0
Tactile	Nets	0	0	0.5	0
Olfactory	*Orco*	0.6	−0.2	−0.6	−0.2
	Antennal removal	2.5	−0.5	1	0.5
	CHC	1.25	0	1	0

### 3.3 Responses of *D. melanogaster* to conspecific and heterospecific rivals following manipulation of auditory cues

We observed a significant effect of the manipulation of auditory cues available to focal *D. melanogaster* males on mating duration (achieved by removing wings from all nonfocal males in “rival” treatments, KW $\chi_{5}^{2}=50.151$, *p *<* *.0001; Figure 2b). Post hoc tests revealed that *D. melanogaster* males did not extend mating duration following exposure to wingless *D. yakuba* “rivals.” The response to similar *D. virilis* exposure was intermediate and not significantly different to either no‐rival males or conspecific rival treatments. There was no difference between the duration of mating seen in response to conspecific males and following exposure to *D. simulans* or *D. pseudoobscura* wingless “rivals.” Indeed, in comparison with unmanipulated responses, the responses of *D. melanogaster* males to *D. simulans* and *D. pseudoobscura* rivals were more than doubled (Table 2).

When hearing‐defective mutant *iav D. melanogaster* males were used as focal males, there was again a highly significant effect of rival treatment on the subsequent mating duration of focal *D. melanogaster* males (KW $\chi_{5}^{2}=35.714$, *p *<* *.0001; Figure 2c). After exposure to *D. yakuba* or *D. virilis, D. melanogaster iav* mating duration was intermediate and not significantly different to either no‐rival or conspecific rival treatments. Exposure of *D. melanogaster iav* males to *D. simulans* and *D. pseudoobscura* rivals resulted in subsequent mating durations that were not significantly different to the conspecific rivals’ response. The difference in median mating duration was increased following exposure to *D. simulans*,* D. yakuba,* and *D. pseudoobscura* males in comparison with the unmanipulated treatments (Table 2). Together, the results suggest that removing auditory cues rendered *D. melanogaster* males significantly more likely to respond to heterospecific males.

### 3.4 Responses of *D. melanogaster* to conspecific and heterospecific rivals following manipulation of tactile cues

When tactile cues were removed by separating males from rivals using porous netting, there was a marginally significant effect of treatment on focal *D. melanogaster* mating duration (KW $\chi_{5}^{2}=13.54$, *p *=* *.019; Figure 2d). Investigation of the standardized median differences to the no‐rival treatment showed that mating duration responses of *D. melanogaster* focal males were either decreased (following exposure to *D. simulans*) or similar to the unmanipulated mating duration responses (Table 2). This suggests that removing tactile cues (mechanosensory or gustatory) reduced the likelihood of males responding to a heterospecific “rival.”

### 3.5 Responses of *D. melanogaster* to conspecific and heterospecific rivals following manipulation of olfactory cues

In olfactory‐defective *Orco* mutant *D. melanogaster* focal males, rival exposure treatment again significantly affected subsequent mating duration (KW $\chi_{5}^{2}=24.072$, *p *=* *.0002; Figure 2e). However, in this experiment, only exposure to a conspecific rival significantly increased mating duration. There was an intermediate response to *D. simulans* and no significant response to *D. yakuba*,* D. pseudoobscura,* or *D. virilis*. Median differences were similar (*D. simulans*) or smaller than observed in the unmanipulated experiments (Table 2).

Following removal of the third antennal segment in the *D. melanogaster* focal males, rival treatment also significantly affected mating duration (KW $\chi_{5}^{2}=20.859$, *p *=* *.0009; Figure 2f). Males exposed to conspecifics and *D. simulans* rivals mated for significantly longer, whereas mating duration after exposure to males of the other species was not significantly different to either no‐rival or conspecific rival treatments. The standardized median differences were increased for *D. simulans, D. pseudoobscura,* and *D. virilis* treatments, but decreased for *D. yakuba* (Table 2).

There was no significant interaction between rival male treatment and *D. melanogaster* CHC treatment (AoD *F*_5, 339_ = 1.918, *p *=* *.091) although a marginally nonsignificant trend for CHC addition to increase mating duration overall (AoD *F*_1, 329_ = 3.379, *p *=* *.067). There was a highly significant effect of rival male treatment (AoD *F*_5, 344_ = 8.754, *p *<* *.0001; Figure 2g). Mating duration was not different to the no‐rival treatment after exposure to *D. yakuba* and *D. virilis*. The response of *D. melanogaster* males to *D. pseudoobscura* rivals was not significantly different to either the no‐rival or conspecific treatments, whereas the response of *D. melanogaster* males to *D. simulans* rivals was not significantly different to that to a conspecific rival. Standardized median responses to *D. yakuba* and *D. virilis* were not different to the unmanipulated responses, but were increased after exposure to *D. simulans* and *D. pseudoobscura* (Table 2).

These findings suggest that the addition of *D. melanogaster* olfactory cues through CHC extracts either rendered males more likely to respond to heterospecifics (*D. simulans* and *D. pseudoobscura*) or had no effect. However, the two manipulations designed to remove olfactory cues did not give equivalent results. *Orco* mutant male responses were comparable to those of unmanipulated wild‐type *D. melanogaster* males, whereas wild‐type males lacking the 3rd antennal segment were more likely to show an increased response to heterospecifics.

## 4 DISCUSSION

The results show that in the absence of manipulations to sensory cues, exposure of *D. melanogaster* males to heterospecifics could elicit significant increases in mating duration, but not to the extent observed following exposure to conspecific rival males. Intriguingly, the extent of *D. melanogaster* responses to heterospecific males did not align with increasing genetic distance between species. Hence, *D. melanogaster* males consistently responded to males of some species with which they never (*D. pseudoobscura*) or rarely (*D. simulans*) hybridize. Manipulation of sensory inputs altered the pattern of *D. melanogaster* mating duration responses to heterospecific males. However, none of the manipulations resulted in *D. melanogaster* males responding to all the other species tested. Where sensory manipulations did have an effect, either through removing information or providing false information (+CHCs), the outcome was generally to increase the extension of mating in response to a heterospecific male. Hence, *D. melanogaster* males that received less accurate information were more likely to *increase* their investment in mating duration, at least toward species to which they had already mounted an intermediate mating duration response. Moreover, different manipulations of the same modality did not necessarily achieve consistent responses, suggesting that these manipulations may have altered sensory information in different ways. In accordance with our previous results (Bretman, Westmancoat et al., 2011), we found no evidence that visual cues are used by *D. melanogaster* males in responding to rivals.

Given an unmanipulated sensory repertoire, we predicted that if *D. melanogaster* were to respond to any heterospecific rival, it would mostly likely be to the closely related *D. simulans*. These species differ in both song parameters (Kawanishi & Watanabe, 1980; Schilcher & Manning, 1975) and CHC components (Jallon & David, 1987). Although *D. melanogaster* males will readily court *D. simulans* females, there is prezygotic isolation between them (Coyne & Orr, 1989). Furthermore, gene expression changes in female *D. melanogaster*, particularly of olfactory and immune‐related genes, are evoked by *D. melanogaster* but not *D. simulans* courtship song (Immonen & Ritchie, 2012). We found that after exposure to *D. simulans*, mating duration of *D. melanogaster* focal males was intermediate between males kept in isolation or exposed to a conspecific rival. Across the four species tested, if the response to rivals reflected phylogenetic relatedness (Tamura et al., 2004), we would expect to see a pattern in which *D. simulans* < *D. yakuba* < *D. pseudoobscura* < *D. virilis*. However, the results showed instead that *D. melanogaster* males (i) never responded to *D. yakuba* or *D. virilis* males by extending mating duration and (ii) exhibited intermediate mating duration responses to *D. pseudoobscura* and *D. simulans* males. The results could be explained by two nonmutually exclusive processes. For species in which sympatry is fairly recent, the pattern could occur if *D. melanogaster* males respond to males of other species that could pose a real sperm competition threat and that show least divergence in terms of sensory cues (i.e., as in the intermediate response to *D. simulans*). However, if discrimination ability is costly, then it should be lost under allopatry (Magurran & Ramnarine, 2004; Wellenreuther et al., 2010) (i.e., as in the intermediate response to *D. pseudoobscura*). It is possible that clades in which males do not respond to conspecifics also fail to elicit rivals’ responses from heterospecifics, a possibility that would be interesting to test further.

Although our sensory manipulations significantly altered responses to heterospecific rivals, the results did not support the idea that a single sensory modality confers information about species identity. No single manipulation “tricked” male *D. melanogaster* into responding to males of all other species. The use of nets to separate males from rivals abolished all responses to heterospecific rivals. This could indicate that removing tactile cues actually increased the discriminatory ability of *D. melanogaster* males. However, it might also indicate that males received insufficient information to mount a response. Further work is needed to distinguish these possibilities. Responses of *D. melanogaster* males to *D. simulans* were generally increased by manipulations of auditory and olfactory cues. The pattern was similar for *D. pseudoobscura*, with the exception of the abolition of a response when focal males carried the *Orco* mutation. Exposure to *D. yakuba* or *D. virilis* rarely elicited even an intermediate response, and in the two instances where this effect did occur, it was via different sensory routes (i.e., hearing‐defective *iav* focal *D. melanogaster* males responding to *D. yakuba*; focal *D. melanogaster* males lacking the 3rd antennal segment responding to *D. virilis*). We did not use combinations of sensory manipulations as this abolishes the response to conspecifics, which would be uninformative for this current study. These findings suggest that multiple traits are used by *D. melanogaster* males to assess species identity in this context.

In *Drosophila*, acoustic, gustatory, tactile, visual, and chemosensory cues have all been implicated in sexual isolation (Cobb & Ferveur, 1996; Greenspan & Ferveur, 2000). Both courtship songs and displays (e.g., Ritchie, Halsey, & Gleason, 1999; Saarikettu, Liimatainen, & Hoikkala, 2005) and CHCs (acting as pheromones) (e.g., Frentiu & Chenoweth, 2010; Rundle, Chenoweth, Doughty, & Blows, 2005) have been identified as targets for sexually‐selected isolating mechanisms and as having driven speciation (Coyne, Crittenden, & Mah, 1994; Etges & Tripodi, 2008; Ritchie et al., 1999). A recent study of *Drosophila athabasca* races, which diverged only 16–20 TYA, suggested that song traits were the driver of isolation and suggested that, for older divergence events, there can be a risk of attributing divergence to traits that may have accumulated postspeciation (Yukilevich, Harvey, Nguyen, Kehlbeck, & Park, 2016). Moreover, multiple manipulations of the same sensory modality might not necessarily fully replicate the cue removed. For example, removal of the 3rd antennal segment is likely to inhibit both olfaction and hearing (Gopfert & Robert, 2002), but may not fully remove either input, as, for example, *Orco* is also expressed in the maxillary palps (Larsson et al., 2004). Likewise, separation by nets may impair both mechanosensory and gustatory signals. We conclude that our findings support our hypothesis that some information about species identity is carried via a multimodal assessment of rivals. However, it is not clear that this information is encoded in one specific sensory cue. It remains to be determined whether *D. simulans* and *D. pseudoobscura* elicit intermediate responses in *D. melanogaster* males due to song or CHC profile similarities.

We assume that the extra investment of responding to heterospecific rivals is costly, based on the finding of shorter survival and greater reproductive senescence in males that repeatedly respond to conspecific rivals (Bretman et al., 2013). However, this should be directly tested. Similarly, we assume that the potential benefits of conspecific responses to rivals are not realized in heterospecific interactions. We did not observe behaviors between males prior to mating, and there are so far scant data on interspecies aggression in *Drosophila*. It would be useful in future work to test for any additional effects on the costs and benefits of rivals’ responses due to aggression or competition for food. Such effects might be mediated in part through differences in body size. However, we note that *D. simulans* and *D. yakuba* are a similar size to *D. melanogaster,* and all are smaller than *D. pseudoobscura* and particularly *D. virilis* (Pitnick, Markow, & Spicer, 1995). Hence, the failure of *D. melanogaster* males to respond to *D. yakuba* or *D. virilis* males seems unlikely to be due to body size‐mediated effects per se.

Our results support the finding that vision plays a negligible role in assessing sperm competition risk, in contrast to the results of Kim et al. (2012). There was also no evidence of differences due to genetic background across Dahomey and Canton‐S strains. Kim et al. (2012) suggested that *D. melanogaster* males respond to *D. simulans* and *D. virilis* as if they are rivals, a pattern that was not found here (i.e., for *D. virilis*, which never responded). In line with our results, a study in *D. pseudoobscura* found vision to be unimportant in responding to rivals (Maguire et al., 2015). In addition, *D. pseudoobscura* males are found not to mount a response to *D. persimilis* rivals (Price et al., 2012). We suggest that the use of a visual cue such as a generalized response to red eyes (Kim et al. (2012) could represent an “evolutionary trap” (Schlaepfer, Runge, & Sherman, 2002), with a high risk of inducing inaccurate, and potentially costly, responses to individuals that cannot pose a sperm competition threat.

## CONFLICT OF INTERESTS

The authors have no competing interests to declare.

## AUTHOR CONTRIBUTIONS

AB and TC conceived and designed the study. AB, JR, and JW collected data. AB analyzed the data. AB, JR, and TC drafted the manuscript.

## DATA ACCESSIBILITY

Data are archived in the DRYAD data repository (https://doi.org/10.5061/dryad.4vs00).
